# Validation of BrainCheck Digital Cognitive Assessments: Test‐Retest Reliability and Concurrent Validity Compared to Traditional Paper‐Based Tests

**DOI:** 10.1002/alz70857_101109

**Published:** 2025-12-24

**Authors:** Duong Huynh, NaZanin Mirshahi, Emilirgh Wong, Mary Patterson, Reza Hosseini Ghomi, Bin Huang

**Affiliations:** ^1^ BrainCheck Inc., Austin, TX, USA; ^2^ BrainBox Solutions Inc., Richmond, VA, USA; ^3^ Frontier Psychiatry, Billings, MT, USA

## Abstract

**Background:**

Digital Cognitive Assessments (DCA) are a promising solution for early dementia detection. BrainCheck has developed an evidence‐based DCA tool, BrainCheck Assess (BC‐Assess), designed to help healthcare providers quickly and reliably evaluate patients with cognitive impairments. Previous studies demonstrated that BC‐Assess effectively distinguishes individuals with normal cognition, mild cognitive impairment, and dementia. This study aims to assess the test‐retest reliability and concurrent validity of BC‐Assess vs traditional paper‐and‐pencil neuropsychological tests.

**Method:**

In collaboration with Brainbox Solutions Inc., 529 participants, who self‐reported as cognitively healthy, were recruited from four US clinical sites after informed consent (NCT04423198). A subset completed BrainCheck assessments twice, with >7 days between tests (test‐retest subset), and another subset completed both BrainCheck assessments and corresponding paper‐and‐pencil tests during the same visit (concurrent validity subset). The BC‐Assess includes assessments evaluating cognitive domains of memory (Immediate/Delayed Recognition), executive function (Stroop), attention and mental flexibility (Trails A/B), and processing speed (Digit Symbol Substitution). These assessments were paired with traditional tests, including the Hopkins Verbal Learning Test (HVLT), Stroop Color Word Test, Trail Making Test, and Wechsler Adult Intelligence Scale (WAIS), respectively. Test‐retest reliability was assessed using intraclass correlation coefficients (ICCs), and Pearson's correlations evaluated concurrent validity between BrainCheck assessments and paper‐and‐pencil tests.

**Result:**

The test‐retest subset included 60 participants (28 females; age range 18‐84 years: M = 48, SD = 18; 30 with ≤12 years education). ICC values for BrainCheck assessments ranged from 0.69‐0.89 (p < .001), indicating moderate to good reliability. For concurrent validity, data from 68 participants (34 females; age range 18‐82 years: M = 48, SD = 18; 33 with ≤ 12 years education) revealed moderate correlations (*r* = 0.31–0.57, *p* < = .01) between BrainCheck assessments and their paper‐based counterparts.

**Conclusion:**

The results demonstrate that BrainCheck offers reliable and valid assessments of cognitive function, comparable to traditional paper‐and‐pencil tests. These findings further validate BC‐Assess as an effective cognitive screening tool.

